# Understanding health literacy in men: a cross-sectional survey

**DOI:** 10.1186/s12889-024-19223-0

**Published:** 2024-07-06

**Authors:** Ruth Mursa, Christopher Patterson, Gemma McErlean, Elizabeth Halcomb

**Affiliations:** 1 School of Nursing, Faculty of Science, Medicine & Health, University of Wollongong, Northfields Ave, Wollongong, NSW 2522 Australia; 2 Health Innovations Research Centre, Faculty of Science, Medicine and Health, University of Wollongong, Wollongong, Northfields Ave, NSW 2522 Australia; 3 Center for Research in Nursing and Health, St George Hospital, Kogarah, NSW 2217 Australia

**Keywords:** Health literacy, Men, Health literacy questionnaire, HLQ

## Abstract

**Background:**

Males have a shorter life expectancy than females. Men are less likely to seek the advice of a health professional or utilise preventive health services and programs. This study seeks to explore health literacy and the characteristics affecting this among Australian men.

**Methods:**

Four hundred and thirty-one adult males engaged with the New South Wales Rural Fire Service, completed an online cross-sectional survey, undertaken from September – November 2022. The survey tool captured demographic data, health status and lifestyle risk characteristics. Health literacy was measured using the 44-item Health Literacy Questionnaire (HLQ). Descriptive statistics, frequencies, percentages, means and standard deviations, were used to describe the sample. Interferential statistics, including the Mann-Whitney U Test and the Kruskal-Wallis Test, were used to explore differences between demographics and HLQ scales.

**Results:**

For the first 5 scales (4-point Likert scale), the lowest score was seen for ‘*Appraisal of health information*’ (Mean 2.81; SD 0.52) and the highest score was seen for ‘*Feeling understood and supported by healthcare providers ’* (*Mean* 3.08; SD 0.64). For the other 4 scales (5-point Likert scale), the lowest score was seen for *‘Navigating the healthcare system’* (Mean 3.74; SD 0.69). The highest score was seen for *‘Understand health information well enough to know what to do’* (Mean 4.10; SD 0.53). Age, income level and living in an urban/rural location were significantly related to health literacy scales.

**Conclusions:**

This study provides new insight into men’s health literacy and the factors impacting it. This knowledge can inform future strategies to promote men’s engagement with health services and preventive care.

## Background

There has been a steady increase in the lifespan of adults in the developed world since the mid-20th century. Internationally, males have a shorter life expectancy than females [1]. In Australia, males live to an average of 81.3 years, while the average female lives to 85.4 years [2]. Life expectancy is impacted by the absence or presence of disease, the complex interactions of a person’s genetic makeup, lifestyle, and environmental factors. In addition, gender shapes all aspects of health and well-being, how illness is experienced, how people manage their health, and their associated health behaviours including help-seeking, and engagement with healthcare services [3].

The specific health conditions that impact the life expectancy of men are well documented globally and include a range of chronic conditions [4]. Indeed the main causes of death in males are, ischaemic heart disease, stroke and chronic obstructive pulmonary disease [5]. Chronic conditions are responsible for approximately 74% of all deaths globally [6]. In Australia, some 49% of men have one or more chronic health conditions and around 75% are overweight or obese [4]. Being overweight is a well-established risk factor for cardiovascular disease, cancer, stroke, diabetes, dementia, asthma and chronic kidney disease [4]. Males are dying earlier than females due to lifestyle factors such as poor diet, smoking, excessive alcohol consumption, and sedentary lifestyles [4]. These lifestyle factors are largely modifiable by behaviour change and quality preventive healthcare. Behaviour change is complex and multifaceted, and health literacy and access to quality healthcare are key contributing factors in being able to make change and work towards better health and improved quality of life.

Health literacy refers to how people access, understand, appraise, and use health information to inform their health and healthcare [7]. It comprises both the individuals’ skills in finding, understanding and using relevant health information to make decisions about health and healthcare, and the health literacy environment, including how infrastructure, policy and practice impact engagement and service use [7]. Health literacy is not just a resource held by the individual, but rather a collective societal responsibility in addressing economic, environmental and social determinants of health. Health literacy provides the means to empower individuals and communities to increase control over their health and enhance health outcomes [8]. Given that an individual’s health-related decisions and subsequent actions are closely related to their level of education and literacy [9], there are strong correlations between health literacy, health behaviours and subsequent health outcomes [10]. Indeed, health literacy is one of the greatest factors influencing a person’s overall health [9]. Adults with low health literacy are less likely to engage in preventive health activities, have higher healthcare utilisation [11], and lower levels of compliance with prescribed medications [12] and advice provided by a clinician [10]. Low health literacy has been associated with smoking, insufficient physical activity and being overweight, as well as lower overall physical and mental health and well-being [13]. Health literacy is also a key component of managing chronic conditions, as the individual requires the skills to facilitate self-management and successfully navigate the complexities of the healthcare system [14].

The role of nurses is central to primary healthcare. Nurses play a pivotal role in healthcare delivery, shaping health policy and improving health outcomes [15]. Nurses are integral in enhancing the health literacy of the patients and their support network as well as assisting them to navigate the increasingly complex health system. The dissemination of health information is empowering to patients and heightens their health literacy [16]. In endeavouring to enhance health literacy, nurses must first assess the patients’ skills to ensure that any health information provided, is effectively communicated at a level appropriate to the individual [17]. Nurses support patients by ensuring they are provided with the information and knowledge to make informed decisions about their health and healthcare journey in the pursuit of optimising health outcomes [16].

Mursa et al. [18] identified that men often do not understand the diversity of care provided by general practice beyond acute care delivery. In addition, men failed to appreciate the delivery of preventive healthcare within general practice and are therefore not utilising this service to its full capacity. Given the importance of understanding men’s engagement with healthcare providers and preventive care it is important to understand the role of health literacy in this cohort [18]. Low levels of health literacy have been identified in males, negatively impacting help-seeking and engagement with healthcare providers [19]. Interestingly, evidence suggests that males working in male-dominated occupations have poorer health literacy than males working in non-male-dominated occupations [20]. This highlights the impact that masculinity has on healthcare engagement, however, research addressing men’s health literacy remains underdeveloped [21].

Therefore, this paper aims to describe health literacy amongst Australian men and explore the characteristics that impact it. The paper reports on the quantitative survey data collected within a mixed methods project exploring Australian men’s help-seeking and engagement with general practice. Other survey data and findings from the subsequent interviews explored different elements of the research problem and are reported elsewhere.

## Methods

A cross-sectional survey was undertaken via REDCap [22] from September – November 2022.

### Participants

Adult males working or volunteering for the New South Wales Rural Fire Service (NSW RFS) were recruited to provide a range of men from diverse socio-economic and educational backgrounds across rural and metropolitan areas. The NSW RFS is the world’s largest volunteer fire service and is made up of both volunteers and employed staff. The community-based service is located in over 150 centres across NSW and is responsible for a variety of services including emergency response, such as fighting fires and managing storm and flood damage as well as bush fire management and mitigation. Office based roles include community engagement and education, training and emergency planning and logistics [23].

### Survey tool

The survey tool was developed based on the current literature. It comprised three sections and combined a validated tool with investigator-developed items. Section one asked about respondent demographics, including age, ethnicity, relationship status and educational background. Section two consisted of health-related questions about current health and self-management, as well as items related to lifestyle risk factors (smoking, nutrition, alcohol, and physical activity) and engagement with health care.

**Fig. 1:**
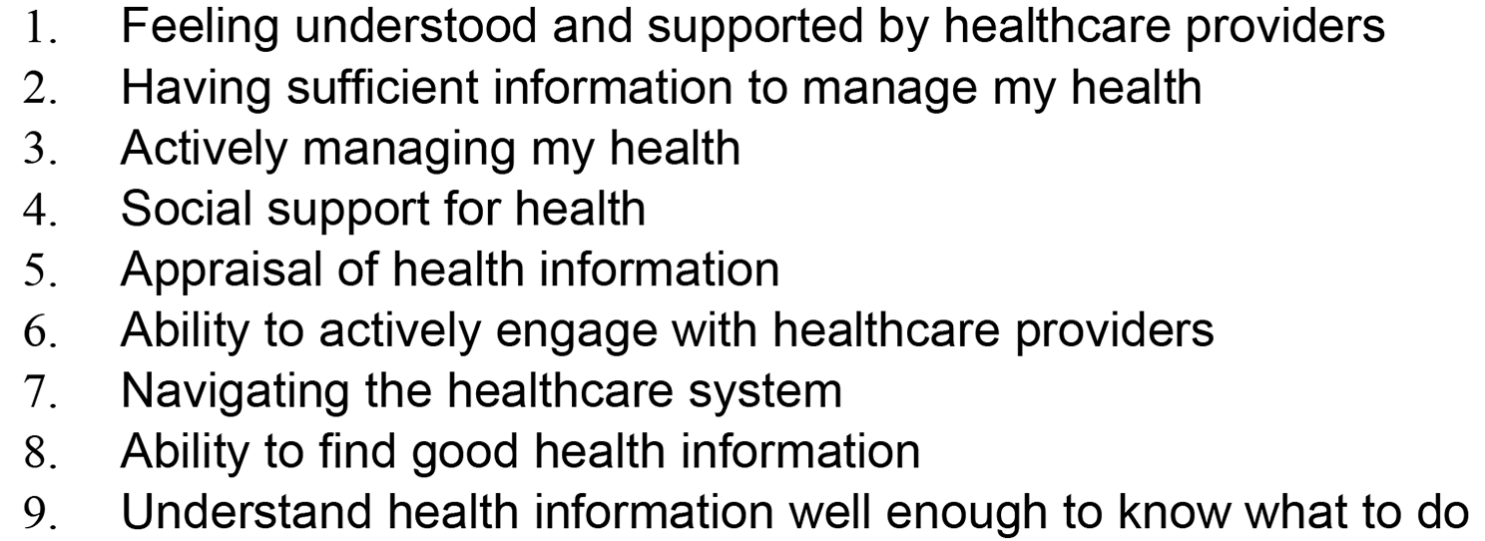
HLQ scales

The final section comprised the Health Literacy Questionnaire (HLQ) [24]. The HLQ consists of 9 scales derived from 44 items rated on either a 4 (scales 1–5; 1 strongly disagree – 4 strongly agree) or a 5-point Likert scale (scales 6 to 9; 1 cannot do or always difficult − 5 always easy) [24] (Fig. 1). The overall HLQ has a Cronbach’s alpha of 0.8, demonstrating good internal consistency [24]. As the survey yielded a large volume of data, this paper focuses on the analysis of the HLQ data (Sect. 3). Other data address a discrete aim about lifestyle risk and so are reported separately.

The survey tool was piloted by three nurse academics, three NSW RFS staff and three community-based men before dissemination to check face and content validity. Minor changes were made to the wording and format of some items based on the feedback received.

### Data collection

Survey information and a link to the online survey were disseminated via a dedicated RFS Facebook page and published in the organisation’s regular e-bulletin. Key contacts within the NSW RFS also shared study information with their contacts and participants were asked to share the information within their networks. The survey was open for 2 months and several reminders were provided via key contacts and social media to promote participation. Four AUD$100 gift vouchers were offered in a prize draw as an incentive to complete the online survey. The STROBE checklist was followed to ensure study quality [25].

### Data analysis

Data were exported from REDCap and analysed using IBM Statistical Package for the Social Sciences (SPSS©)(Version 28.0). Data cleaning was undertaken to identify missing values. Those responses missing over half of the items or duplicate responses were excluded.

The HLQ scoring syntax, rules and interpretation matrix were used to guide analysis. For scales with 4–5 items (scales 1–6, 8 and 9), up to 2 missing values were imputed, with responses excluded if responses for more than 2 item were missing from that scale. Similarly, for the scale with 6 items (scale 7), up to 3 missing values were imputed, with responses missing more than 3 items excluded on that scale. The expectation maximization (EM) algorithm was used to impute missing HLQ item scores.

Descriptive statistics, frequencies, percentages, means and standard deviations, were used to describe the sample. Interferential statistics, including the Mann-Whitney U Test and the Kruskal-Wallis Test, were used to explore differences between demographics and HLQ scales [26]. A *p*-value < 0.05 indicated statistical significance.

## Results

### Respondent characteristics

Seventy-four (14.7%) of the 505 responses received were excluded, leaving 431 responses (85.3%) for analysis (Table 1). Most respondents (87.7%; *n* = 378) were born in Australia. Respondents’ mean age was 52.67 years (range 18–90 years; SD 16). Three-quarters of respondents (75.9%; *n* = 327) volunteered for the NSW RFS and 16.2% (*n* = 70) were both an employee and volunteer. Respondents’ locality was spread across both rural (52.9%; *n* = 228) and urban (47.1%; *n* = 203) areas. There was diversity in the highest level of education, with 20.6% (*n* = 89) having a high school education, 43.6% (*n* = 188) having completed vocational education, and 35.7% (*n* = 154) with a tertiary qualification. Most respondents (85.6%; *n* = 369) were in a relationship, with 80.0% (*n* = 345) of respondents living with a partner.

**Table 1:** Respondent demographics

Characteristic	*n*	%
**Age** (Mean 52.67 years, SD 16, range 18–90)		
≤ 39 years	99	23
40–64 years	214	49.7
≥ 65 years	118	27.4
**Relationship with RFS**		
Volunteer	327	75.9
Employee	34	7.9
Employee and Volunteer	70	16.2
**Location**		
Rural	228	52.9
Urban	203	47.1
**Born in Australia**		
No	53	12.3
Yes	378	87.7
**Ethnic group**		
Australian	393	91.2
Indigenous Australian or Torres Strait Islander	7	1.6
Other	31	7.2
**Relationship status**		
Single	62	14.4
Partner/de facto/married	369	85.6
**Highest education**		
High school	89	20.6
Vocational education/training	188	43.6
University	154	35.7
**Average household income**		
≤$45,000	59	13.7
$45,001 - $180,000	284	65.9
>$180,000	79	18.3
Missing	9	2.1

### HLQ scores

Table 2 shows the mean score for each HLQ scale. All items in scales 1–5 were completed by all respondents, however, 9 respondents had missing items from scales 6–9. For the first 5 scales (4-point Likert scale), the lowest score was seen for ‘*Appraisal of health information*’ (Mean 2.81) and the highest score was seen for ‘*Feeling understood and supported by healthcare providers’* (*Mean* 3.08). For the scales scored on a 5-point Likert scale, the lowest score was seen for *‘Navigating the healthcare system’* (Mean 3.74). The highest score was seen for *‘Understand health information well enough to know what to do’* (Mean 4.10).

**Table 2:** HLQ scores

HLQ Scale	Mean	SD
	Range 1–4	
Feeling understood and supported by healthcare providers	3.08	0.64
Having sufficient information	3.04	0.49
Actively managing health	2.84	0.57
Social support for health	2.96	0.55
Appraisal of health information	2.81	0.52
	Range 1–5	
Understand health information well enough to know what to do	4.10	0.53
Active engagement with healthcare providers	3.91	0.69
Ability to find good health information	3.91	0.54
Navigating the healthcare system	3.74	0.69

There were several significant differences between HLQ scales and demographic groups (Table 3). *‘Feeling understood by healthcare providers’* was significantly impacted by age (all groups *p* < 0.01) and income (≤$45,000 versus >$180,000; *p* = 0.003). Those who were older and had lower incomes scored significantly higher and so were more likely to have an established relationship with a healthcare provider whom they trust.

**Table 3:** Association between HLQ scores and demographics

		Feeling understood & supported by HCP	Have sufficient information to manage health	Actively managing my health	Social support for health	Appraisal of health information	Ability to actively engage with HCP	Navigating the healthcare system	Ability to find good health information	Understand health information well enough to know what to do
		Mean (SE)	Mean (SE)	Mean (SE)	Mean (SE)	Mean (SE)	Mean (SE)	Mean (SE)	Mean (SE)	Mean (SE)
Age	< 39 yrs	2.85 (0.07)	3.02 (0.05)	2.77 (0.07)	2.93 (0.06)	2.79 (0.05)	3.79 (0.07)	3.67 (0.07)	3.87 (0.06)	4.06 (0.05)
	40–64 yrs	3.06 (0.04)	3.01 (0.03)	2.80 (0.04)	2.88 (0.04)	2.77 (0.03)	3.84 (0.05)	3.67 (0.05)	3.88 (0.04)	4.06 (0.04)
	≥ 65 yrs	3.32 (0.05)	3.12 (0.04)	2.97 (0.05)	3.13 (0.04)	2.89 (0.05)	4.13 (0.06)	3.93 (0.06)	3.98 (0.05)	4.19 (0.04)
	*p*-value	< 0.001*	0.071	0.007*	0.001*	0.202	< 0.001*	0.001*	0.241	0.130
Location	Rural/regional	3.07 (0.04)	2.99 (0.03)	2.80 (0.04)	2.91 (0.04)	2.77 (0.03)	3.89 (0.04)	3.71 (0.04)	3.89 (0.03)	4.06 (0.03)
	Urban/metro	3.11 (0.05)	3.10 (0.04)	2.89 (0.04)	3.02 (0.04)	2.85 (0.04)	3.93 (0.05)	3.77 (0.05)	3.94 (0.04)	4.14 (0.04)
	*p*-value	0.421	0.022*	0.160	0.039*	0.128	0.314	0.233	0.257	0.104
Relationship status	Single	3.01 (0.09)	3.06 (0.07)	2.92 (0.08)	2.67 (0.08)	2.83 (0.06)	3.93 (0.09)	3.77 (0.08)	3.97 (0.06)	4.09 (0.07)
	In relationship	3.10 (0.03)	3.04 (0.02)	2.83 (0.03)	3.01 (0.03)	2.81 (0.03)	3.91 (0.04)	3.73 (0.04)	3.90 (0.03)	4.10 (0.03)
	*p*-value	0.386	0.927	0.201	< 0.001*	0.802	0.630	0.950	0.415	0.891
Born in Australia	No	3.02 (0.10)	2.99 (0.08)	2.79 (0.09)	2.95 (0.09)	2.83 (0.07)	3.88 (0.09)	3.73 (0.09)	4.00 (0.06)	4.15 (0.06)
	Yes	3.10 (0.03)	3.05 (0.02)	2.85 (0.03)	2.96 (0.03)	2.81 (0.03)	3.91 (0.04)	3.74 (0.04)	3. 90(0.03)	4.09 (0.03)
	*p*-value	0.534	0.891	0.529	0.734	0.834	0.577	0.967	0.132	0.599
Education	High school	3.11 (0.07)	3.04 (0.06)	2. 90 (0.07)	2.98 (0.07)	2.76 (0.06)	3.91 (0.08)	3.79 (0.07)	3.83 (0.05)	4.02 (0.04)
	Vocational	3.07 (0.05)	3.01 (0.03)	2.77 (0.04)	2.92 (0.04)	2.75 (0.03)	3.89 (0.053)	3.73 (0.05)	3.88 (0.04)	4.05 (0.04)
	University	3.10 (0.05)	3.08 (0.04)	2.90 (0.05)	3.00 (0.04)	2.90 (0.04)	3.93 (0.05)	3.72 (0.06)	3.98 (0.04)	4.19 (0.04)
	*p*-value	0.819	0.485	0.104	0.554	0.011*	0.967	0.831	0.064	0.046*
Income	≤$45,000	3.32 (0.07)	3.08 (0.07)	2.95 (0.07)	2.97 (0.07)	2.84 (0.08)	4.09 (0.08)	3.79 (0.10)	3.84 (0.08)	4.12 (0.06)
	$45,001-$180,000	3.08 (0.04)	3.03 (0.03)	2.83 (0.03)	2.96 (0.03)	2.80 (0.03)	3.89 (0.04)	3.73 (0.04)	3.89 (0.03)	4.07 (0.03)
	>$180,000	2.91 (0.08)	3.03 (0.06)	2.76 (0.06)	2.90 (0.06)	2.80 (0.05)	3.80 (0.08)	3.70 (0.08)	4.00 (0.06)	4.15 (0.06)
	*p*-value	0.003*	0.647	0.353	0.671	0.960	0.050*	0.925	0.071	0.580

*statistical significance

Similarly, *‘Ability to actively engage with healthcare providers’* was also significantly impacted by age (all groups *p* < 0.001) and income (≤$45,000 versus >$180,000; *p* = 0.050). Older respondents and those with lower incomes were more proactive about their health and felt more in control with healthcare providers.

Age alone significantly impacted the scales, *‘Actively managing my health’* (*p* = 0.007) and *‘Navigating the healthcare system’* (*p* = 0.001). Respondents in the ≤ 39 years (*p* = 0.029) and 40–64 years (*p* = 0.010) age groups both scored significantly lower than the ≥ 65 years group in terms of actively managing their health. Being older indicated a more proactive approach to the individual’s healthcare journey. Similarly, both ≤ 39 years (*p* = 0.014) and 40–64 years (*p* = 0.002) age groups scored significantly less than the ≥ 65 years group in terms of *‘Navigating the healthcare system’*. Whereas younger respondents were less able to advocate on their own behalf, older respondents were significantly more likely to find services and support to meet their health needs.

Several demographic characteristics significantly impacted *‘Social support for health’*. Being older (40–64 years versus ≥ 65 years *p* = 0.001), living in an urban location (*p* = 0.039) and being in a relationship (*p* < 0.001) saw respondents significantly more likely to feel they have a support network.

Having *‘Sufficient information to manage health’* was impacted by the respondent’s location (*p* = 0.022), with rural respondents having greater gaps in their knowledge and insufficient information to make decisions and manage their health than urban respondents.

Unsurprisingly, respondents’ highest level of education significantly impacted both *‘Appraisal of health information’* (*p* = 0.011) and *‘Understand health information well enough to know what to do’* (*p* = 0.046). Respondents with higher levels of education were more likely to report being able to identify reliable sources of health information and understand all written health information. No demographic factors significantly impacted respondents’ *‘Ability to find good health information’* (*p* > 0.005).

## Discussion

This study has explored the health literacy of a diverse group of Australian men. It has identified several characteristics that impact their skills, knowledge, motivation, and capacity to access, understand, appraise, and apply information to make effective decisions about their health and healthcare. Health literacy is a topic of global relevance, understanding the challenges faced by specific population groups, such as men, is important for nurses as it can inform future strategies to promote health literacy and uptake of preventive care.

The Australian healthcare system is one of the most comprehensive in the world and is made up of both a public and private system. The healthcare workforce comprises of doctors, nurses and midwives, allied health, Indigenous health workers, dentists and support staff [27]. The health system is underpinned by Medicare which pays rebates for medical services provided by health professionals [27]. When Australian residents attend a general practice consultation, part of the consultation fee will be covered by Medicare, however increasingly, an additional out of pocket fee may be required [28]. Nurses working in primary healthcare settings including general practice are integral in providing not only acute care but also in contributing in providing health promotion, health education, disease prevention and the management of chronic diseases [29].

Health literacy impacts help-seeking and engagement with healthcare providers and is integral in supporting and maintaining the health and well-being of the individual and their community [30]. Lower levels of health literacy are more often associated with people on lower incomes [31, 32], as people with higher incomes tend to have better access to health information and resources [33]. This leads to an assumption that people on lower incomes access health services less frequently or have poorer relationships with healthcare providers. However, this study identified that those on lower incomes were more likely to have an established relationship with and felt more in control with healthcare providers and were more proactive about their health. This finding is supported by other studies using the HLQ that reported a similar association [34, 35]. Such a finding is a reminder that assumptions should not be made about low-income earners as this characteristic does not necessarily equate to poor engagement with health services. Further research to explore the nature of these relationships would help to identify how they can be supported.

This study revealed that age is an important factor in health literacy. While older respondents were significantly more likely to find services and support to meet their health needs, younger respondents were less able to advocate on their own behalf. Findings by Zurynski et al. [36], Bo et al. [37] and Australian Bureau of Statistics [38] similarly reported that younger respondents experienced poorer health literacy compared to older people. They suggested that an established relationship with a healthcare provider, and more experience navigating the healthcare system, strengthened older people’s health literacy capabilities. In addition, younger men may lack understanding of where to find quality health information, resources, or services [39]. This highlights an opportunity for nurses to intervene to assist younger men in building their capacity earlier in their life course to enhance help-seeking and engagement with preventive care. Such intervention could improve well-being and health outcomes.

Rural respondents in this study described having insufficient information to make decisions and manage their health. Gaps in health literacy between urban and rural people have been previously recognised [40]. Accessing current, accurate and relevant health information may be challenging in rural/remote communities where consistent, accessible healthcare services and resources may be limited [41]. The reliance of accessing essential information on financial, educational and health via the internet is greater for residents in rural communities [42]. However, given the growing reliance on technology, and the increasing availability of credible online resources, it might have been assumed that the availability of health information has been addressed. This highlights the need for nurses to ensure that health information is readily accessible regardless of location and provided in a format that is appropriate for the target audience. The findings of this study are a reminder that more needs to be done to ensure that credible health information is widely available regardless of geographical location. Given their significant roles in rural locations, nurses are in a prime position to assess the health needs of their local community and implement interventions to enhance health literacy [43].

Beyond information needs, this study also demonstrated that rural respondents expressed a greater need for social support. People living in rural/remote communities are more likely to experience social isolation, and this has a detrimental impact on their overall health including both their physical and mental well-being [44]. The changing face of rural communities has resulted in declining rural populations with an associated loss of local business, industry, and services including health [45]. Social interaction is imperative in maintaining a sense of well-being [45]. Social isolation, loneliness, relationship breakdowns together with sociocultural norms of self-reliance, masculinity and stoicism detrimentally impacts the health of men in rural communities [46]. Having good social support networks can be protective for health and positively impact health and well-being [47]. Social support is linked to improved self-care [48], and a supportive environment encourages positive lifestyle choices [49]. There is a need for nurses to provide support and deliver public health programs that facilitate social connectivity and enhance health engagement and health outcomes for residents in rural communities. Our findings highlight that more needs to be done to address the impact of rurality on health literacy to reduce the gap between rural and urban health outcomes.

### Limitations

Our survey has several limitations. Firstly, the cross-sectional design limits findings to associations, rather than causality. In addition, we used purposive sampling to recruit men from the NSW RFS, and therefore have an inherent degree of research bias. The NSW RFS provided a novel means to access a group of men with diverse educational, socio-economic and physical characteristics. However, men from diverse ethnic and cultural backgrounds were not well represented in the survey and care should be taken in the generalisation of findings. The survey was only offered online, thus potentially excluding participants with lower levels of technological literacy. However, the diversity of respondents provides confidence in the sample distribution. As the survey was completed during the COVID-19 pandemic, there is an inherent degree of research bias due to ramifications of restrictions previously placed on social interactions and healthcare engagement. An additional limitation includes the self-reported nature of the survey, increasing the potential of self-reporting bias and yielding variable scores.

## Conclusions

The findings of the study have provided new insights into the health literacy of men by demonstrating the significance of key characteristics that impact health literacy. Considering the impact of age, income and rurality in the development of future health literacy programs will ensure that such programs best meet the needs of the men who are their focus. In particular, programs need to focus on building capacity in younger men and enhancing access to health information and social support for those living in rural areas. Nurses are integral in building and supporting the health literacy of healthcare consumers but require ongoing support to equip them with the capacity and the means to engage and deliver such programs. Addressing these areas has the potential for the largest gains in health literacy, engagement and health outcomes.

## Data Availability

The data the support the findings of this study are available from the corresponding author upon reasonable request.

## Abbreviations

HLQ: Health Literacy Questionnaire
NSW RFS: New South Wales Rural Fire Service
HCP: Healthcare Providers
